# Supplementary material for: ProteinShader: illustrative rendering of macromolecules
Source: BMC Struct Biol. 2009 Mar 30;9:19. doi: 10.1186/1472-6807-9-19 (PMC2672931; doi:10.1186/1472-6807-9-19)
Supplement: Additional file 1 — ProteinShader program without source code. This compressed file contains the complete ProteinShader program including associated libraries, but no source code. A README.txt file gives an overview of the ProteinShader distribution, and the index.html file in the help subdirectory has directions on getting started with the program as well as a set of tutorials. [file 1472-6807-9-19-S1.zip › ProteinShader-beta-0_9_4-binary/help/api/org/proteinshader/structure/exceptions/BondLengthException.html]

BondLengthException (ProteinShader API)


|  |  |  |
| --- | --- | --- |
| **Overview** \| **Package** \| **Class** \| **Use** \| **Tree** \| **Deprecated** \| **Index** \| **Help** | |  |
| PREV CLASS   **NEXT CLASS** | **FRAMES**    **NO FRAMES**     **All Classes** |  |
| SUMMARY: NESTED \| FIELD \| CONSTR \| METHOD | DETAIL: FIELD \| CONSTR \| METHOD |  |


---


## org.proteinshader.structure.exceptions Class BondLengthException

```
java.lang.Object
  java.lang.Throwable
      java.lang.Exception
          org.proteinshader.structure.exceptions.BondLengthException
```

**All Implemented Interfaces:**: Serializable

---

``` public class BondLengthException extends Exception ```

Can be used to report that a Bond is unacceptably short or long.

**See Also:**: Serialized Form

---

**Constructor Summary**

`BondLengthException(double calculatedLength,
double expectedLength,
double toleranceFactor,
AtomEnum srcAtomType,
AtomEnum dstAtomType,
int srcAtomSerialNo,
int dstAtomSerialNo)`
  
          Sets the default message to "A Bond length was unacceptable".

`BondLengthException(String message,
double calculatedLength,
double expectedLength,
double toleranceFactor,
AtomEnum srcAtomType,
AtomEnum dstAtomType,
int srcAtomSerialNo,
int dstAtomSerialNo)`
  
          The message given this constructor can be retrieved using
getMessage().


| **Method Summary** | |
| --- | --- |
| `double` | `getCalculatedLength()`             Returns the calculated Bond length in angstroms. |
| `int` | `getDstAtomSerialNo()`             Returns the destination Atom PDB serial number. |
| `AtomEnum` | `getDstAtomType()`             Returns the destination Atom type as an AtomEnum. |
| `double` | `getExpectedLength()`             Returns the expected Bond length in angstroms. |
| `int` | `getSrcAtomSerialNo()`             Returns the source Atom PDB serial number. |
| `AtomEnum` | `getSrcAtomType()`             Returns the source Atom type as an AtomEnum. |
| `double` | `getToleranceFactor()`             Returns the tolerance factor used to determine that the calulated length was too far from the expected length. |

**Methods inherited from class java.lang.Throwable**

`fillInStackTrace, getCause, getLocalizedMessage, getMessage, getStackTrace, initCause, printStackTrace, printStackTrace, printStackTrace, setStackTrace, toString`

**Methods inherited from class java.lang.Object**

`clone, equals, finalize, getClass, hashCode, notify, notifyAll, wait, wait, wait`

**Constructor Detail**

### BondLengthException

```
public BondLengthException(double calculatedLength,
                           double expectedLength,
                           double toleranceFactor,
                           AtomEnum srcAtomType,
                           AtomEnum dstAtomType,
                           int srcAtomSerialNo,
                           int dstAtomSerialNo)
```

:   Sets the default message to "A Bond length was unacceptable".
    This message can be retrieved using getMessage().

    **Parameters:**: `calculatedLength` - the calculated bond length in angstroms.: `expectedLength` - the expected bond length in angstroms.: `toleranceFactor` - the tolerance factor for the length.: `srcAtomType` - the source Atom type.: `dstAtomType` - the destination Atom type.: `srcAtomSerialNo` - the source atom PDB serial number.: `dstAtomSerialNo` - the destination Atom PDB serial number.

---


### BondLengthException

```
public BondLengthException(String message,
                           double calculatedLength,
                           double expectedLength,
                           double toleranceFactor,
                           AtomEnum srcAtomType,
                           AtomEnum dstAtomType,
                           int srcAtomSerialNo,
                           int dstAtomSerialNo)
```

:   The message given this constructor can be retrieved using
    getMessage().

    **Parameters:**: `message` - an error message.: `calculatedLength` - the calculated bonc length in angstroms.: `expectedLength` - the expected bond length in angstroms.: `toleranceFactor` - the tolerance factor for the length.: `srcAtomType` - the source Atom type.: `dstAtomType` - the destination Atom type.: `srcAtomSerialNo` - the source Atom PDB serial number.: `dstAtomSerialNo` - the destination Atom PDB serial number.


**Method Detail**

### getCalculatedLength

```
public double getCalculatedLength()
```

:   Returns the calculated Bond length in angstroms.

    :   **Returns:**: The calculated Bond length.

---


### getExpectedLength

```
public double getExpectedLength()
```

:   Returns the expected Bond length in angstroms.

    :   **Returns:**: The expected Bond length.

---


### getToleranceFactor

```
public double getToleranceFactor()
```

:   Returns the tolerance factor used to determine that the calulated
    length was too far from the expected length. For example, if the
    tolerance factor is 0.15, then the calculated Bond length is only
    allowed to be 15 percent longer or shorter than the expected
    length.

    :   **Returns:**: The tolerance factor as a number between 0 and 1.

---


### getSrcAtomType

```
public AtomEnum getSrcAtomType()
```

:   Returns the source Atom type as an AtomEnum.

    :   **Returns:**: The source Atom type.

---


### getDstAtomType

```
public AtomEnum getDstAtomType()
```

:   Returns the destination Atom type as an AtomEnum.

    :   **Returns:**: The destination Atom type.

---


### getSrcAtomSerialNo

```
public int getSrcAtomSerialNo()
```

:   Returns the source Atom PDB serial number.

    :   **Returns:**: The source Atom serial number.

---


### getDstAtomSerialNo

```
public int getDstAtomSerialNo()
```

:   Returns the destination Atom PDB serial number.

    :   **Returns:**: The destination Atom serial number.


---


|  |  |  |
| --- | --- | --- |
| **Overview** \| **Package** \| **Class** \| **Use** \| **Tree** \| **Deprecated** \| **Index** \| **Help** | |  |
| PREV CLASS   **NEXT CLASS** | **FRAMES**    **NO FRAMES**     **All Classes** |  |
| SUMMARY: NESTED \| FIELD \| CONSTR \| METHOD | DETAIL: FIELD \| CONSTR \| METHOD |  |


---

# *Copyright © 2007-2008*
